# A Case in Practice

**Published:** 1885-04

**Authors:** 


### A CASE IN PRACTICE.

In the March number of this journal "Modern Dentist" asks what shall be done with devitalized teeth, filled according to the practice of long ago, when the canals were left open and a hole was bored beneath the gum for drainage. An instructive case of this kind lately fell into our hands. Twenty years ago a dead second superior molar was filled without special treatment. Abscess supervened, and an opening was made beneath the gum. During all the long intervening period caries had not attacked the cavity made by the drill, nor had there been absorption of the alveolus and recession of the gum sufficient to bring the drainage hole to the surface. On the contrary, the continued periostial irritation had resulted in exostoses, or hypertrophies, not only upon the roots of the molars, but on the alveoli as well. Large nodules could be plainly seen and felt upon both the buccal and lingual walls, and the tissue presented a peculiarly rough and ragged appearance. The adjoining wisdom tooth, which was extensively decayed, was with difficulty extracted, and subsequently the offending second molar was removed. The roots of both presented unusually large hypertrophies. The patient stated that, while there had been no pain of an acute character, there had never been a time when the region was free from a vague uneasiness, at times approaching a dull, sub-acute distress. It is not pleasant for an intelligent person to reflect upon the condition of that mouth during this long period. The discharge of pus must have been exceedingly disagreeable, to say nothing of the exposure to danger of pyæmia from its possible absorption, or the hazard of necrosis from an acute inflammation.

Such treatment at the present day would, by most competent dentists, be called malpractice. When these cases come for treatment, it is necessary first to obtain direct access to the pulp chamber and the nerve canals, by an opening through the crown. If there be a large proximal filling this may be removed, and an approach thus secured. The buccal opening should then be permanently closed, and the usual treatment resorted to until the discharge wholly ceases and the tooth is rendered thoroughly aseptic, when it will usually be found that all the disturbed territory has returned to a healthy condition.

